# Chemokine-derived oncolytic peptide induces immunogenic cancer cell death and significantly suppresses tumor growth

**DOI:** 10.1038/s41420-024-01932-5

**Published:** 2024-04-02

**Authors:** Natsuki Furukawa, Wendy Yang, Alex R. Chao, Akash Patil, Adam C. Mirando, Niranjan B. Pandey, Aleksander S. Popel

**Affiliations:** 1 Department of Biomedical Engineering, Johns Hopkins University School of Medicine, Baltimore, MD USA; 2 The Sidney Kimmel Comprehensive Cancer Center, Johns Hopkins University School of Medicine, Baltimore, MD USA

**Keywords:** Cell death, Breast cancer

## Abstract

Chemokinostatin-1 (CKS1) is a 24-mer peptide originally discovered as an anti-angiogenic peptide derived from the CXCL1 chemokine. Here, we demonstrate that CKS1 acts not only as an anti-angiogenic peptide but also as an oncolytic peptide due to its structural and physical properties. CKS1 induced both necrotic and apoptotic cell death specifically in cancer cells while showing minimal toxicity in non-cancerous cells. Mechanistically, CKS1 disrupted the cell membrane of cancer cells quickly after treatment and activated the apoptotic pathway at later time points. Furthermore, immunogenic molecules were released from CKS1-treated cells, indicating that CKS1 induces immunogenic cell death. CKS1 effectively suppressed tumor growth in vivo. Collectively, these data demonstrate that CKS1 functions as an oncolytic peptide and has a therapeutic potential to treat cancer.

## Introduction

Currently, most drugs used for cancer treatment are small molecules or antibodies [1]. In general, small molecules show low affinity and low specificity towards their targets which causes undesired side effects. Although therapeutic antibodies are well suited to interfere with protein–protein interactions, poor tissue penetration due to their large size is a prominent drawback. High production costs also limit their availability to patients [2]. Peptides possess unique properties that can overcome the disadvantages of these therapies [3, 4]. Similar to antibodies, peptides can bind to their target protein with high affinity and high specificity. Moreover, peptides are small and can penetrate tissues better than antibodies. Peptides are synthesized by chemical reactions which makes the production costs significantly lower than antibodies that require the usage of biological materials and protein purification. Therefore, peptide therapeutics have the potential to be effective and low-cost cancer treatments.

Chemokinostatin-1 (CKS1) is a 24-mer peptide originally discovered as an anti-angiogenic peptide. Karagiannis and Popel developed a computational method to identify anti-angiogenic sequences contained in the human proteome and discovered common sequences conserved within protein domains contained in angiogenesis-related proteins [5]. CKS1 is derived from a sequence shared in the CXC chemokine family. The anti-angiogenic property of CKS1 was verified experimentally [6], and its efficacy in suppressing tumor growth was demonstrated in triple-negative breast cancer (TNBC) [7] and glioma [8] models.

Oncolytic peptides selectively induce cell death in cancer cells. They typically have an amphipathic structure and a net positive charge [9]. Oncolytic peptides interact with the negatively charged molecules on the cell membrane, such as phosphatidylserine and glycosylated proteins, via electrostatic interactions. Once the concentration of the peptide on the surface of the cancer cells exceeds a threshold, the peptides disrupt the cell membrane and eventually induce cell death. Three major models have been proposed to explain the cell penetration step [10]. In the barrel pore model, the peptides span through the membrane to form a pore. The toroidal pore model proposes that the peptides interact with the lipids in the cell membrane to form a pore composed of peptides and lipids. In the carpet model, the peptides cover the surface of the cell membrane and carve out the lipids by forming micelle-like particles.

The specificity of oncolytic peptides for cancer cells over non-cancerous cells is explained by the differences in the components of the cell membrane. In healthy cells, flippases and floppases function to maintain the asymmetric distribution of phospholipids. Neutral phospholipids such as sphingomyelin and phosphatidylcholine are mostly located in the outer layer of the cell membrane, while negatively charged phospholipids such as phosphatidylserine and phosphatidylinositol are sequestered in the inner layer of the cell membrane. Due to this asymmetric distribution of phospholipids, the cell surface of healthy cells is neutral [11]. However, the machinery to maintain the asymmetric distribution is frequently impaired in cancer cells, leading to the exposure of negatively charged phospholipids. Accordingly, the cell surface of cancer cells is commonly more negatively charged compared to healthy cells [12]. A high level of lactate production due to the propensity of cancer cells to upregulate glycolysis also contributes to the negative charge of the cancer cell membrane [13].

Since oncolytic peptides are positively charged, they preferentially bind to the cell surface of cancer cells [14]. As a proof of concept, Iwasaki et al. demonstrated that cell lines with higher expression of phosphatidylserine were more sensitive to oncolytic peptides [15]. Moreover, the fluidity of the cell membrane also determines the sensitivity to oncolytic peptides. Cholesterol in the cell membrane decreases the fluidity of the cell membrane and makes it difficult for the oncolytic peptides to insert into the lipid bilayer [14]. Some cancer cell types exhibit higher membrane fluidity due to the decreased amount of cholesterol in the membrane, which leads to higher sensitivity to oncolytic peptides [12].

In addition to the disruption of the cell membrane, disruption of the organelles may also be important for the activity of oncolytic peptides. LTX-315, a 9-mer oncolytic peptide, has been shown to accumulate in the mitochondria and cause mitochondrial outer membrane permeabilization [16, 17]. Treatment with LTX-315 leads to the release of cytochrome *c* and the generation of reactive oxygen species (ROS). Importantly, inhibition of mitochondrial activity with a high concentration of carbonyl cyanide m-chlorophenyl hydrazone, an inhibitor of oxidative phosphorylation, decreased the cytotoxicity of LTX-315. Another oncolytic peptide, LTX-401, accumulates in the Golgi membranes and destroys the Golgi apparatus [18]. Although the molecular mechanism of how the disruption of the Golgi apparatus leads to cell death remains unclear, inhibition of protein transport into the Golgi apparatus using Brefeldin A, led to decreased cytotoxic activity of LTX-401. Despite LTX-315 and LTX-401 both being amphipathic peptides with membrane-disrupting capability, LTX-315 accumulates in the mitochondria but not in the Golgi apparatus, whereas LTX-401 localizes mostly in the Golgi apparatus and the cytosol, and not in the mitochondria [18]. Wodlej et al. compared the localization of R-DIM-P-LF11-322, which is an oncolytic peptide that interacts specifically with cancer cells, and DIM-LF11-318, which is an oncolytic peptide that also induces cell death of non-cancerous cells [19]. R-DIM-P-LF11-322 accumulated in the Golgi apparatus and induced swelling of the mitochondria and eventually apoptosis. On the other hand, the type of cell death caused by DIM-LF11-318 was mainly necrosis caused by the rupturing of the cell membrane. These data indicate that each oncolytic peptide exhibits a unique subcellular localization which may affect the mechanism of cell death caused by each peptide.

Cell death caused by oncolytic peptides has been reported to induce the release of immunogenic molecules from the cells [20–22]. Oncolytic peptides contribute to the treatment of cancer not only by killing cancer cells but also by stimulating anti-tumoral immunity. LTX-315 has been shown to synergize with anti-CTLA4 antibodies in a mouse sarcoma model [23]. In a small clinical trial, LTX-315 treatment led to increased expression of immune-related genes, increased T-cell clone expansion in blood, and increased infiltration of immune cells into tumors in melanoma, sarcoma, and breast cancer [24]. Currently, several clinical trials evaluating the efficacy of LTX-315 monotherapy and combinations of LTX-315 with immunotherapies are being conducted.

Although CKS1 was originally identified as an anti-angiogenic peptide, its predicted structural properties indicated potential oncolytic activity. Here we use several biochemical and imaging assays to confirm cancer-specific membrane disruption and subsequent apoptosis while minimally impacting normal cells. This mechanism of cell death was also found to release the immunogenic molecules, suggesting possible synergy with immune modulation. Finally, anti-tumor activity was demonstrated in two mouse models. Together with the previously discovered anti-angiogenic properties, the oncolytic activities uncovered in these studies emphasize the potential of CKS1 as a promising cancer therapeutic.

## Results

### CKS1 has a suitable structure to be an oncolytic peptide

**Fig. 1:**
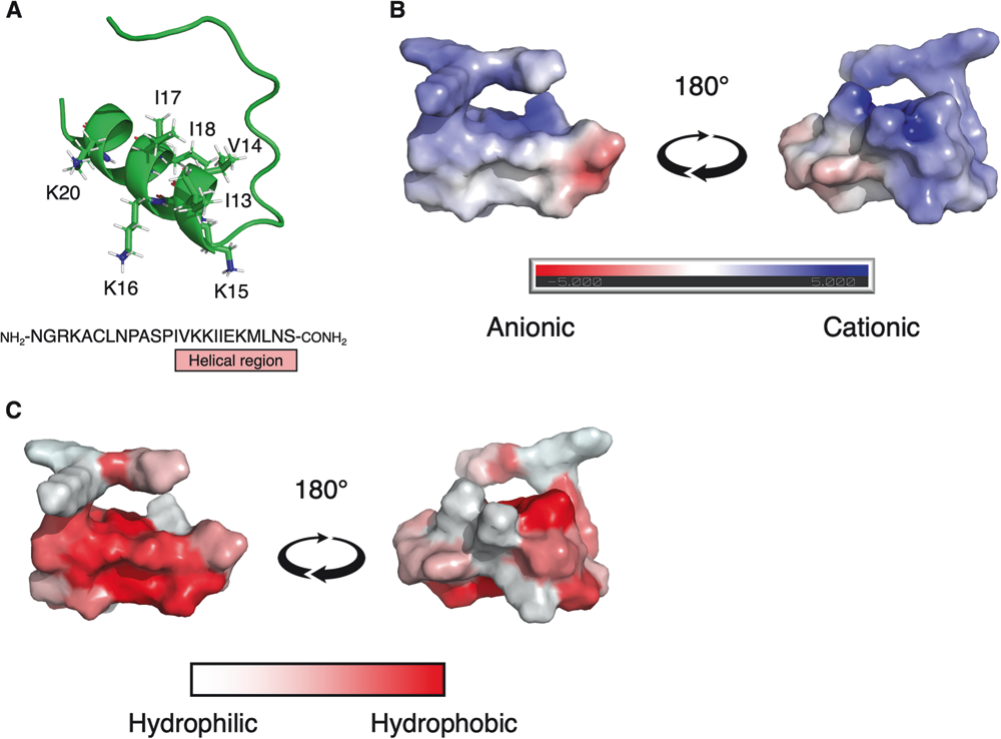
The structure and physical properties of CKS1. **A** The structure of CKS1 predicted by AlphaFold 2. **B** The electrostatic potential of the surface of CKS1 was predicted by the APBS electrostatics plugin in Pymol. Blue regions are the positively charged regions and red regions are the negatively charged regions. **C** The hydrophobic regions of CKS1 are visualized as red and the hydrophilic regions are visualized as white.

To examine the structure of CKS1 (NGRKACLNPASPIVKKIIEKMLNS), we predicted the structure using ColabFold [25]. CKS1 was predicted to contain an unstructured N-terminal region and a C-terminal α-helix (Fig. 1A). Importantly, CKS1 contains several basic amino acids that make the net charge positive (Fig. 1B). The charged amino acids are concentrated on one side of the helix, and the other side of the helix is formed by hydrophobic amino acids (Fig. 1C). The cationic amphipathic helical structure of CKS1 is consistent with that of oncolytic peptides such as LTX-315 and LTX-401 [26, 27]. We further predicted whether CKS1 would be an oncolytic peptide using ACPred, a support vector machine-based machine-learning model [28]. As a result, CKS1 was predicted to be an oncolytic peptide with a probability of 94.9%. These findings indicated that CKS1 could function as an oncolytic peptide.

### CKS1 induces rapid cell death of cancer cells

**Fig. 2:**
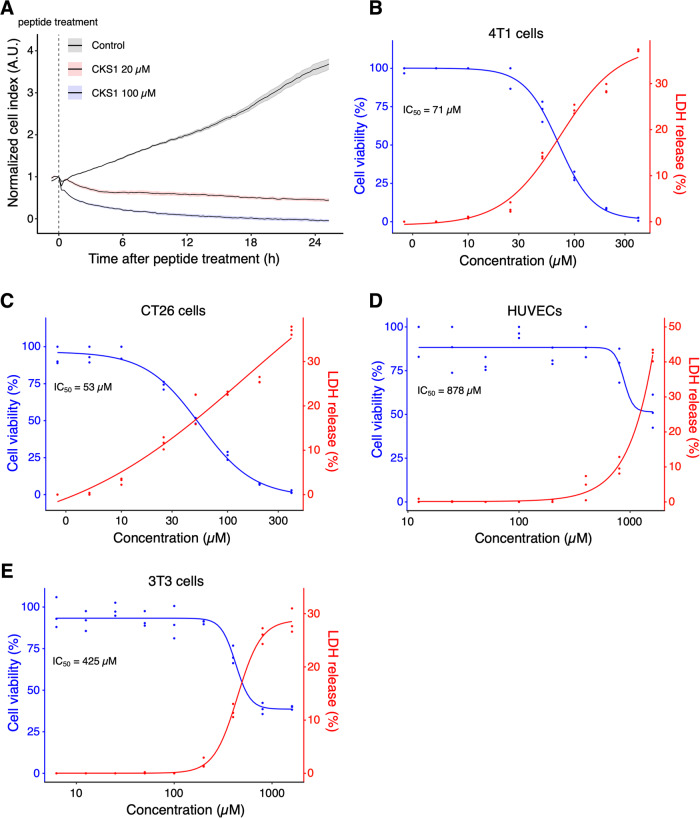
CKS1 induces rapid cell death in multiple cancer cell lines but not in non-cancerous cell lines. **A** Cell viability of 4T1 cells after CKS1 treatment was measured by a real-time cell analyzer (RTCA). The vertical, dotted line indicates the time of peptide addition, and solid lines and the shaded areas show the mean and the standard error of the technical replicates, respectively. Representative of *N* = 3. **B**–**E** The cell viability and the release of LDH were measured after the cells were treated with the indicated concentration of CKS1 for 6 h. The blue data points and the red data points show the cell viability and the release of LDH, respectively. The release of LDH in the supernatant was measured by the CyQUANT LDH cytotoxicity assay and was normalized to the amount of LDH released from cells treated with lysis buffer. The curves show the dose-response curves fitted to the observed data. Each data point shows the technical replicate of each experiment. Representative of *N* = 3 independent experiments.

To determine whether CKS1 functions as an oncolytic peptide, we monitored the cell viability of 4T1 murine TNBC cells after CKS1 treatment using an xCELLigence real-time cell analyzer (RTCA) (Agilent). In this method, the binding of cells to gold electrodes is detected as electrical impedance and displayed as arbitrary units of cell index in real time. Cell death, as suggested by a reduction in cell index, was observed within 1 h of treatment and was dose-dependent (Fig. 2A). We further evaluated the cytotoxic effect of CKS1 by cell viability assay and lactate dehydrogenase (LDH) assay. Cell viability assay was conducted using alamarBlue, which is a blue dye that turns red in response to cellular metabolic reduction. LDH is a cytosolic enzyme that is released outside the cells when the cell membrane is damaged. Cell viability of 4T1 cells decreased in a dose-dependent manner and the half-maximal inhibitory concentration (IC50) was 71 µM ± 5.3 µM (Fig. 2B). Cells treated with CKS1 released significant amounts of LDH showing that CKS1 induces damage in the cell membrane of 4T1 cells. The cytotoxicity of CKS1 was not limited to 4T1 cells, and CKS1 was found to be effective in several murine and human cancer cell lines: CT26 murine colon carcinoma cells with an IC50 of 53 µM ± 9.9 µM (Fig. 2C), human osteosarcoma U2OS cells, and murine fibrosarcoma MCA205 cells (Fig. S1A, B). Importantly, CKS1 induced cell death of NIH/3T3 normal fibroblasts and human umbilical vein endothelial cells (HUVECs) when the concentration was significantly higher than the concentration needed to induce cell death in cancer cells (Fig. 2D, E). At a concentration in which CKS1 did not induce LDH release, CKS1 significantly attenuated cell proliferation of HUVECs as demonstrated by the inhibition of BrdU incorporation (Fig. S1C). These results indicate that the reported anti-angiogenic property is separate from the cell death-inducing capacity of CKS1.

### CKS1 rapidly disrupts the cell membrane of cancer cells

**Fig. 3:**
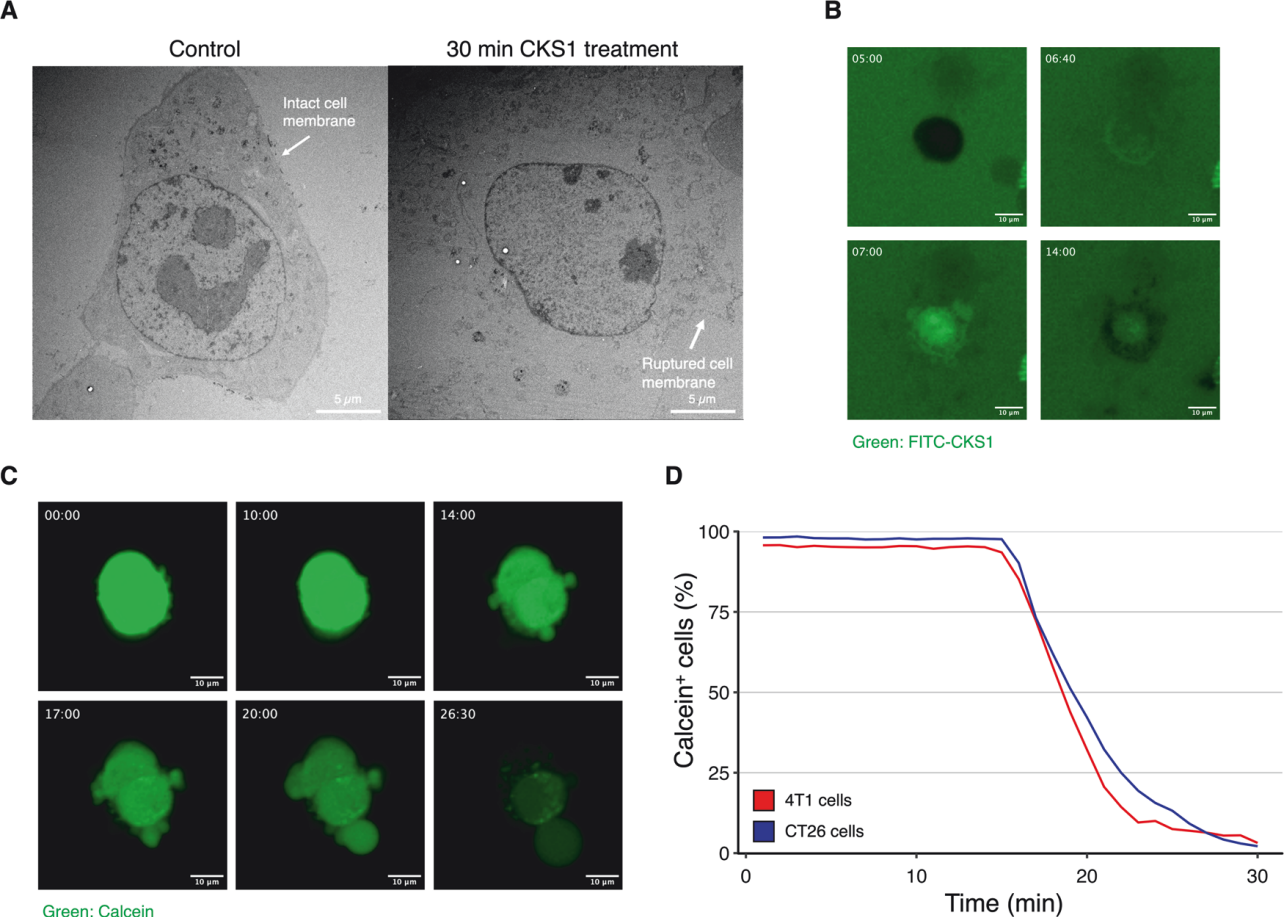
CKS1 rapidly disrupts the cell membrane of cancer cells. **A** 4T1 cells were treated with water or 100 µM CKS1 for 30 min and the detailed structure of the cell was observed using transmission electron microscopy. The cell membrane of 4T1 cells was disrupted after 30 min of CKS1-treatment. **B** 4T1 cells were treated with 100 µM FITC-CKS1 (green) and monitored by fluorescence live cell imaging. The timestamp indicates the minutes after cells were treated with the peptide. Representative of *N* = 3. **C** 4T1 cells were stained with calcein-AM (green) and were treated with 100 µM CKS1. The timestamp indicates the minutes after cells were treated with the peptides. **D** The ratio of cells maintaining calcein in the cytosol at each time point was calculated based on the live cell imaging. Representative of *N* = 3.

To identify the mechanism of cell death caused by CKS1, we observed the morphology of the cells after CKS1 treatment using electron microscopy. The cell membrane—but not the nuclear membrane—of 4T1 cells treated with CKS1 was ruptured after 30 min (Fig. 3A). We attempted to clarify whether the cell membrane damage was induced by intrinsic cellular machinery or by physical damage caused by the peptides. Pretreatment with the pan-caspase inhibitor Z-VAD, RIPK1 inhibitor necrostatin-1 stable (Nec-1s), or ferrostatin-1 (Fer-1) did not inhibit the release of LDH, indicating that the release of LDH by CKS1 does not require these programmed cell death pathways (Fig. S2A, B).

To observe the interactions between the peptide and cancer cells, we produced CKS1 tagged with fluorescein isothiocyanate (FITC) and observed its movement after adding it to the cells (Fig. 3B and Supplemental Video 1 and 2). FITC-CKS1 first accumulated on the cell surface which is seen as a ring around the cell. Next, the peptides entered the cells. A similar phenomenon was observed when CT26 cells were treated with FITC-CKS1 (Supplemental Video 3). To further clarify the events leading to cell death, we stained the cytosol of 4T1 cells using calcein-AM and treated them with CKS1 (Fig. 3C and Supplemental Video 4 and 5). After CKS1 treatment, there was an influx of the surrounding media into the cells. The influx caused swelling of the cells and blebbing to occur. The blebbing eventually bursts, leading to the release of cytosolic components into the extracellular space. By quantifying the fraction of cells containing calcein after CKS1 treatment, we found that the cytosol of cells treated with CKS1 was released within 15–30 min of CKS1 treatment and that the cell membrane of nearly all cells was eventually damaged (Fig. 3D). Consistent with the findings from above, pretreatment with Z-VAD did not inhibit nor slow down the release of calcein (Fig. S2C).

### CKS1 diminishes mitochondrial membrane potential and activates the apoptotic pathway at later time points

**Fig. 4:**
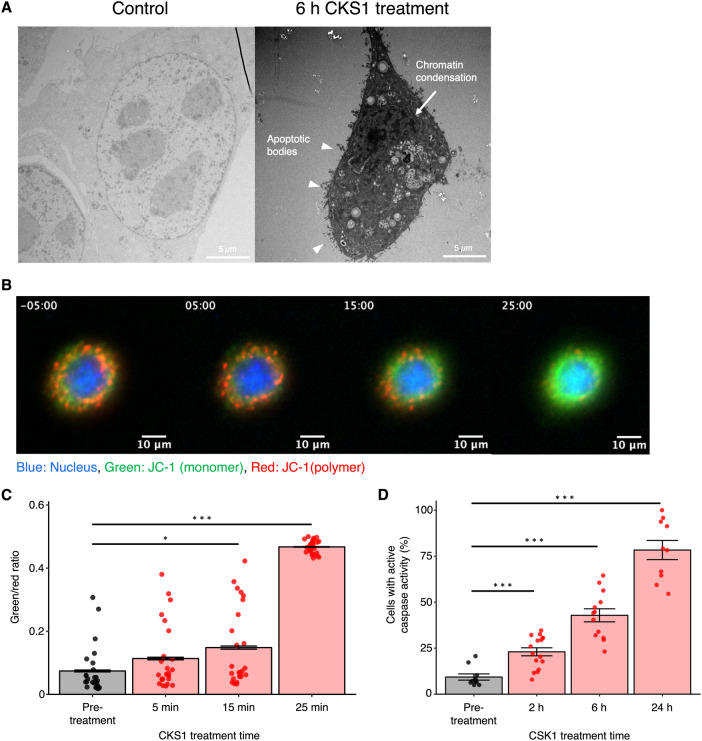
CKS1 diminishes mitochondrial membrane potential and activates the apoptotic pathway. **A** 4T1 cells were treated with water or 100 µM CKS1 for 6 h and the detailed structure of the cell was observed using transmission electron microscopy. Overall shrinkage of the cell, collapse of the nucleus, chromatin condensation (arrow), and apoptotic blebbing (triangle) indicate that the cells underwent apoptosis. **B** Fluorescence live cell imaging of 4T1 cells stained with JC-1 and treated with 100 µM CKS1. The timestamp indicates the minutes after CKS1 treatment (negative value indicates pre-treatment). Green indicates the monomer form of JC-1, and red indicates the polymer form of JC-1. **C** Twenty-five cells were randomly selected from the video of figure A (supplemental video 5) and the ratio between the green intensity and the red intensity was calculated. Means ± SEM, representative of *N* = 3 independent experiments, Dunnett’s test (*p < 0.05, ***p < 0.005). **D** At least ten fields of view were acquired for each time point and the fraction of cells with red intensity in the nucleus (cells with active caspase activity) was calculated. Means ± SEM, representative of *N* = 3 independent experiments, Dunnett’s test (*p < 0.05, ***p < 0.005).

When 4T1 cells were treated with CKS1 for 6 h, we observed cells with apoptotic morphology (Fig. 4A). The volume of the cells shrank, the nucleus collapsed, the chromatin condensed, and apoptotic blebbing occurred. These observations indicate that CKS1 induces caspase-independent necrotic cell death by disrupting the cell membrane at early time points and induces apoptosis at later time points after CKS1 treatment.

We hypothesized that CKS1 first ruptures the cell membrane then enters the cells, disrupts the function of the mitochondria, and activates the apoptotic pathway. To test this hypothesis, we stained 4T1 cells with JC-1. JC-1 is a cationic dye that accumulates in the mitochondria. In intact mitochondria, when the mitochondrial membrane potential is high, JC-1 forms aggregates that emit red fluorescence. When the mitochondrial membrane potential is low, JC-1 exists as monomers that emit green fluorescence. Therefore, the ratio of green and red fluorescence emitted by the two forms of JC-1 functions as an indicator of the mitochondrial membrane potential. We observed a rapid switch of JC-1 emitted fluorescence from red to green when 4T1 cells were treated with CKS1 (Fig. 4B, C and Supplemental Video 6 and 7).

We next quantified the activity of caspase-3 in the lysate of cells treated with CKS1. Caspase-3 was not activated after 30 min of CKS1 but was activated after 4 h of CKS1 treatment (Fig. S3A). This indicates that the activation of the apoptotic pathway is a secondary phenomenon that happens after the cell membrane is ruptured. To observe the fraction of cells with active caspases, we conducted live cell imaging using CellEvent Caspase-3/7 detection reagent, which is a dye that stains the nucleus when caspase-3 or caspase-7 is activated. The fraction of cells with activated caspases increased over time (Fig. 4D and Fig S3B). Note that since cancer cells detach after cell death, it was impossible to accurately assess the number of cells with activated caspases. Our data indicate that there are at least two fates of the cells treated with CKS1; cells that undergo severe damage and detach from the dish and cells that stay attached to the dish and undergo apoptosis.

These data collectively suggest that in addition to cell death caused by the rupturing of the cell membrane, disruption of the mitochondrial activity and the subsequent activation of the apoptotic pathway may contribute to the cell death caused by CKS1.

### The helical region of CKS1 drives the oncolytic activity

**Fig. 5:**
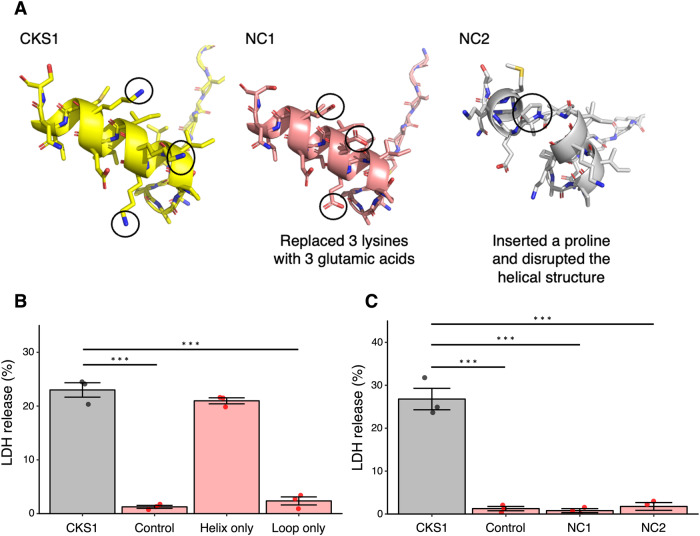
The cationic helical structure of CKS1 is important for its activity. **A** The structure of CKS1, NC1, and NC2. **B**, **C** The release of LDH in the supernatant was measured after treating 4T1 cells with 100 µM of the indicated peptides for 6 h. Data were normalized to cells treated with lysis buffer. Means ± SEM, *N* = 3, Dunnett’s test (***p < 0.005).

Our motivation to test the oncolytic activity of CKS1 was the observation that CKS1 possesses the characteristic amphipathic, positively charged α-helix. To examine whether these features are responsible for the oncolytic activity, we produced a peptide that only contains the C-terminal helical region (IVKKIIEKMLNS) and a peptide that contains the N-terminal loop region (NGRKACLNPASP). Furthermore, we produced two mutant peptides, NC1 and NC2 (Fig. 5A). In NC1, the three lysines that form the hydrophilic side of CKS1 were replaced with glutamic acids. Since lysines are positively charged and glutamic acids are negatively charged, these mutations make the peptide anionic rather than cationic. In NC2, a single proline was inserted in the middle of the α-helix of CKS1. Since prolines destabilize α-helices, the helical structure of CKS1 would be expected to be disrupted in NC2. As expected, the peptide with just the helical region of CKS1 induced LDH release from 4T1 cells, while the peptide with only the loop region of CKS1 did not (Fig. 5B). Although the loop region was not directly involved in the oncolytic activity, it may enhance the oncolytic activity of CKS1 by stabilizing the structure or increasing the solubility. For example, the peptide with only the helical region was poorly soluble in water, while CKS1 showed high solubility in water (data not shown). NC1 and NC2 did not show oncolytic activity, demonstrating that the cationic property and the helical property are both essential for the oncolytic activity of CKS1 (Fig. 5C).

### CKS1 induces immunogenic cell death

**Fig. 6:**
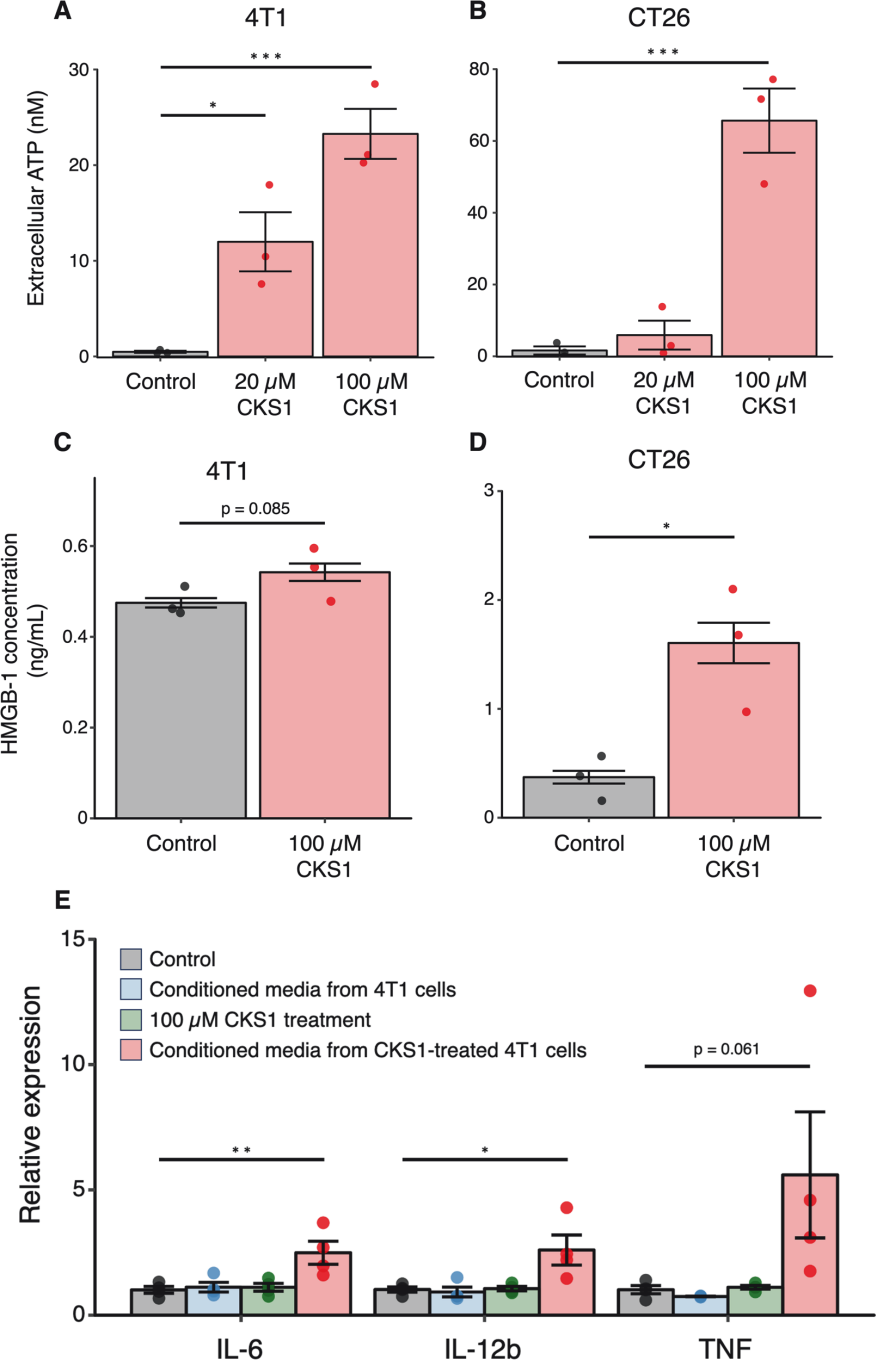
CKS1 induces the release of immunogenic molecules. **A**, **B** Cells were treated with 20 µM or 100 µM CKS1 for 30 min and the amount of ATP released in the culture media was quantified. Means ± SEM, *N* = 3, Dunnet’s test (*p < 0.05, ***p < 0.005). **C**, **D** Cells were treated with 100 µM CKS1 for 6 h and the amount of HMGB-1 released in the culture media was quantified. Means ± SEM, *N* = 3, Welch’s *t*-test (*p < 0.05). **E** DC2.4 cells were treated with either complete media (control), conditioned media from 4T1 cells, 100 µM CKS1, or conditioned media from 4T1 cells treated with 100 µM CKS1. The relative expression level normalized to the expression level in the control condition is shown. Means ± SEM, *N* = 4, Dunnet’s test (*p < 0.05, **p < 0.01).

The release of damage-associated molecular patterns (DAMPs) such as ATP and HMGB-1 is a representative characteristic of immunogenic cell death [29]. To determine whether cell death caused by CKS1 is immunogenic, we evaluated the amount of ATP and HMGB-1 released in the culture media upon CKS1 treatment of 4T1 and CT26 cancer cells. During the process of immunogenic cell death, ATP is released before cell death, while HMGB-1 is released after cell death [30]. Therefore, we observed the release of ATP 30 min after CKS1 treatment, and the release of HMGB-1 6 h after treatment. We observed a significant release of ATP from both cell lines (Fig. 6A, B). Although the release of HMGB-1 from 4T1 cells treated with CKS1 was weak, CT26 cells treated with CKS1 showed a significant release of HMGB-1 (Fig. 6C, D).

We further evaluated the immunogenicity of CKS1 by testing whether the molecules released by 4T1 cells during CKS1 treatment can activate DC2.4 murine dendritic cells. In this experiment, 4T1 cells were treated with CKS1, and the conditioned media was collected. DC2.4 cells were treated with the conditioned media for 2 h and the expression level of three proinflammatory cytokines, TNF, IL-6, and IL-12b was measured. Since the conditioned media also contains CKS1 and molecules released from live cells, we tested appropriate control conditions to test the contribution of these factors. Treatment with conditioned media from CKS1-treated 4T1 cells significantly increased the expression level of the cytokines, while treatment of CKS1 nor the treatment with the conditioned media from live 4T1 cells did not (Fig. 6E). These data indicate that CKS1 induces immunogenic cell death.

### CKS1 induces necrosis and inhibits tumor growth in vivo

**Fig. 7:**
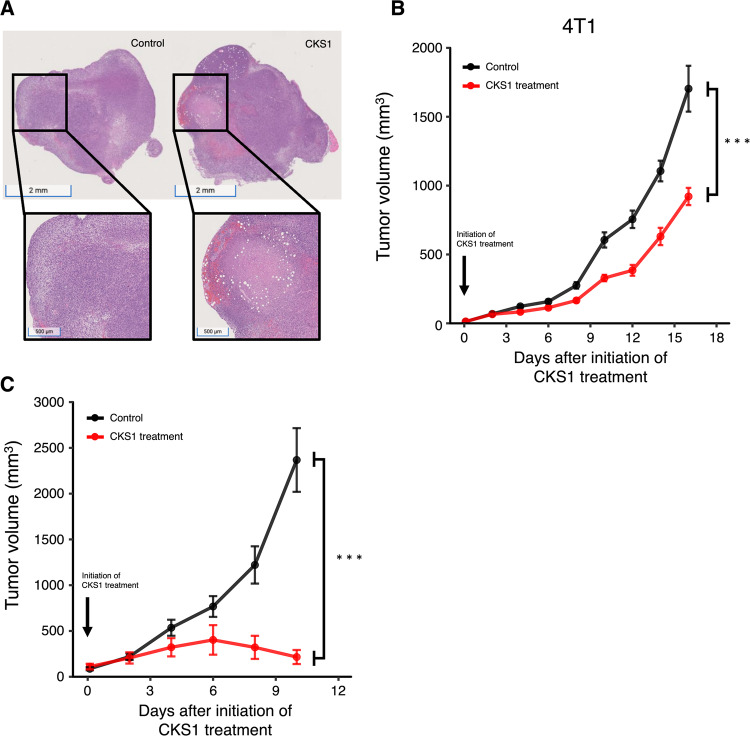
CKS1 induces necrosis and suppresses tumor growth in vivo. **A** CKS1 was injected intratumorally in established 4T1 tumors. After 24 h, tumors were excised and were stained with H&E stain. The white area indicates necrosis caused by CKS1. Representative of *N* = 3. **B**, **C** The tumor volume of 4T1 tumors and CT26 tumors were recorded. Fourty milligram/kg CKS1 was applied daily starting on day 7. Means ± SEM, 4T1 control *N* = 15, 4T1 CKS1 treatment *N* = 15, CT26 control *N* = 10, CT26 CKS1 treatment *N* = 10. Two-way repeated-measures ANOVA was performed to determine the significance of CKS1 treatment (***p < 0.005).

To evaluate whether CKS1 can induce cancer cell death in vivo, we injected CKS1 intratumorally into established 4T1 tumors. The intratumoral injection was chosen to ensure the delivery of peptides to the tumors. Necrosis was observed in tumors 24 h after CKS1 treatment (Fig. 7A). Furthermore, the growth of both 4T1 tumors and CT26 tumors was inhibited by CKS1 treatment (Fig. 7B, C).

## Discussion

Our data demonstrate that CKS1 induces cell death in cancer cells with low cytotoxicity to healthy cells. We observed that the cell membrane of cancer cells is ruptured by CKS1 within 30 min of peptide treatment. When the CKS1 concentration was significantly higher than the IC_50_ value, the cell membrane of nearly 100% of the cancer cells was damaged. At later time points, we observed cells with activated caspases that underwent apoptosis. The majority of the cells detached after cell death, however, a fraction of the cells were attached to the dish with an apoptotic morphology. Based on these observations, our model of CKS1-induced cell death is summarized in Fig. S4. First, the peptides accumulate on the cell surface of the cancer cells and create pores in the cell membrane. If the cell membrane damage is severe, the cells are lysed. These cells die before the activation of the apoptotic pathway and detach from the dish. If the cell membrane damage is moderate, cells somehow survive potentially via the activation of the membrane repair mechanisms [31]. However, CKS1 enters the cells via the pores formed and targets the mitochondria to activate the apoptotic pathway. These cells eventually undergo apoptosis.

Although membrane rupture is a common phenomenon caused by oncolytic peptides, the induction of apoptosis is only seen in a few oncolytic peptides. For example, LTX-315 failed to stimulate caspase-3 activity, while inducing a necrotic cell death [20]. The factors that determine whether a peptide induces apoptosis or not are not fully known. Our data indicate that the mitochondrial membrane potential rapidly diminishes after peptide treatment. Although the mechanism of how CKS1 treatment affects the mitochondria remains unanswered, the malfunctioning of mitochondria caused by CKS1 may be the trigger of apoptotic cell death.

Our data showed that cells treated with CKS1 released immunogenic molecules. Anti-angiogenic agents improve the quality of the vasculature in the tumor (vascular normalization), leading to the alleviation of an immunosuppressive environment caused by hypoxia and acidosis and infiltration of immune effector cells [32]. Therefore, CKS1 may induce anti-cancer immunity by two means: release of immunogenic molecules and vascular normalization. The synergy between oncolytic peptides and cancer immunotherapy has been demonstrated for LTX-315 [23, 24, 33]. The potential of CKS1 as an immunotherapy has not been discussed in this study and further work would be required to determine the impact of CKS1 on anti-cancer immunity.

Our data show that CT26 tumors were highly sensitive to CKS1, while 4T1 tumors showed a relatively modest response to CKS1. The cell viability assay and LDH assay demonstrated that CT26 cells are more sensitive to CKS1, which may explain the difference of CKS1 efficacy in these models.

The obstacles to translating CKS1 to the clinic are the expected poor stability in vivo because of its all-natural amino acid composition and the need for a drug delivery system. To avoid these problems, we utilized intratumoral injection in the current study. Intratumoral injection can ensure the accurate delivery of the drug to the tumor and can limit the risk of systemic side effects since it is a local administration method. Because of these benefits, intratumoral injection is adopted in multiple immunotherapies, such as immune receptor agonists and oncolytic viral therapies. Currently, more than 20 neoadjuvant clinical trials using these intratumoral immune stimulatory agents and their combinations are ongoing [34]. Intratumoral injection is typically employed for solid tumors exposed on the surface of the body such as breast cancer and melanoma. Therefore, these cancer types may be good indications for treatment with CKS1.

In summary, CKS1 is a unique peptide that exerts anti-angiogenic and oncolytic activity to suppress tumor growth. Our experimental data indicate that cell membrane rupture and induction of the apoptotic pathway contribute to cell death. Since the oncolytic activity is not dependent on specific markers in cancer cells, CKS1 has the potential to be applied to a wide range of cancer types.

## Materials and methods

### Peptides

CKS1 (NGRKACLNPASPIVKKIIEKMLNS), FITC-CKS1 (CKS1 with FITC tagged at the N-terminus), the helical region of CKS1 (IVKKIIEKMLNS), the loop region of CKS1 (NGRKACLNPASP), NC1 (NGRKACLNPASPIVEEIIEEMLNS), and NC2 (NGRKACLNPASPIVKKIIPEKMLNS) were synthesized by Genscript (Piscataway, NJ) using a solid-phase peptide synthesis method. There is a free amine at the N-termini of all these peptides while they were all amidated at the C-terminus. The purity was >90% as verified by HPLC and MS analyses. CKS1, the fragments of CKS1, and FITC-CKS1 were solubilized in water. NC1 and NC2 were solubilized in DMSO.

### Cell culture

4T1 murine mammary carcinoma cells (CRL-2539), CT26 murine colon carcinoma cells (CRL-2638), U2OS human osteosarcoma cells (HTB-96), and NIH/3T3 normal murine fibroblast cells (CRL-1658) were purchased from the American Type Culture Collection (Manassas, VA). MCA205 murine fibrosarcoma cells (SCC173) and DC2.4 murine dendritic cells (SCC142) were purchased from MilliporeSigma (Burlington, MA). Cell lines were mycoplasma-tested before being used. 4T1 cells, MCA205 cells, and DC2.4 cells were propagated in RPMI 1640 (Corning, Corning, NY) supplemented with 10% FBS (MilliporeSigma). NIH/3T3 cells and U2OS cells were propagated in DMEM (Thermo Fisher Scientific, Waltham, MA) supplemented with 10% FBS. HUVECs were propagated in the EGM-2 bulletkit (LONZA, Basel, Switzerland). All cell lines were grown in T75 tissue culture flasks (Sarstedt, Nümbrecht, Germany) under standard culture conditions of 37 °C and 5% CO_2_.

### Prediction of the peptide structures

The structures of the peptides were predicted by the protein structure prediction software AlphaFold 2 [35]. The prediction of the protein structure was done using the ColabFold platform [25] with the default parameters and was visualized by Pymol [36]. The electrostatic property of the peptides was calculated using the APBS plugin in Pymol.

### Prediction of oncolytic peptide

Prediction of whether CKS1 functions as an oncolytic peptide was done using ACPred (http://codes.bio/acpred/) [28]. The sequence of CKS1 was used as the input and the default parameter was used for the prediction.

### Animal models

The protocols used in this study were approved by the Institutional Care and Use Committee at Johns Hopkins Medical Institutions. Four- to eight-week-old female Balb/c mice were obtained from Charles River (Wilmington, MA). For the 4T1 triple breast cancer model, 2.5 × 10^4^ 4T1 cells were injected into the first mammary fat pad of each mouse. For the CT26 colon carcinoma model, 1.0 × 10^6^ CT26 cells were injected subcutaneously in the left flank. The sample size was determined so that the final distribution would be close to normal distribution. After 1 week, the animals were randomized into control and CKS1 treatment. During the randomization, the mice were kept in a cage covered with cloth so that the investigator could not see the tumor size. We started daily intratumoral treatment with 40 mg/kg CKS1. For the control group, the matching volume of water was treated intratumorally. The tumor size was measured by using a caliper, and the volume was calculated by using the formula 0.52 × (length) × (width)^2^.

### Real-time cell viability assay

Three thousand cells were seeded in the wells of an xCELLigence RTCA E-plate 16 (Agilent, Santa Clara, CA). After the cells adhered to the plate, the media was replaced with serum-free media, and the cells were treated with CKS1 at the indicated concentrations. The viability of the cells was monitored using the xCELLigence RTCA analyzer (Agilent).

### LDH assay

Ten thousand cells were seeded in a 96-well plate and incubated overnight. The cells were washed once, and the medium was replaced with serum-free media. Cells were treated with CKS1 for the indicated time and concentration. CyQUANT LDH cytotoxicity assay (Thermo Fisher Scientific) was used following the manufacturer’s instructions to measure the release of LDH. LDH release was normalized to the amount of LDH released from cells treated with lysis buffer.

### Cell viability assay

Ten thousand cells were seeded in a 96-well plate and incubated overnight. The cells were washed once, and the media was replaced with serum-free media. After peptide treatment for the indicated time and concentration, the cells were washed once and 90 µL of fresh serum-free media was added to each well. Ten microliter of alamarBlue (Bio-Rad Laboratories, Hercules, CA, BUF012A) was added and the cells were incubated for 3 h. After a 1:5 dilution of the supernatant, the fluorescence (excitation/emission = 570 nm/610 nm) was measured using a plate reader. Finally, the cell viability was calculated by the following formula: cell viability (%) = (fluorescence from each sample – fluorescence from lysis buffer treated cells)/(fluorescence from non-treated cells – fluorescence from lysis buffer treated cells).

### BrdU assay

One thousands cells were seeded in wells of a 96-well plate. The cells were treated with the indicated concentration of CKS1 immediately after seeding. Forty-eight hours later, 5-bromo-2’-deoxyuridine (BrdU) was added to each well. Twenty four hours later, BrdU incorporation was quantified using the BrdU cell proliferation assay kit (Cell Signaling Technology, Danvers, MA) following the manufacturer’s instructions.

### Electron microscopy

Samples were fixed in 2.5% glutaraldehyde, 3 mM MgCl_2_ in 0.1 M sodium cacodylate buffer, pH 7.2 overnight at 4 °C. After buffer rinse, samples were postfixed in 2% osmium tetroxide in 0.1 M sodium cacodylate for at least 1 h (no more than two) on ice in the dark. After osmium, samples were rinsed in 0.1 M Maleate buffer (pH 6.2), followed by uranyl acetate in 0.1 M Maleate (0.22 µm filtered, 1 h, dark), dehydrated in a graded series of ethanol and embedded in Epon (PolySci) resin. Samples were polymerized at 60 °C overnight. Thin sections, 60–90 nm, were cut with a diamond knife on a Leica UCT ultramicrotome and picked up with 2 × 1 mm Formvar copper slot grids. Grids were stained with 2% uranyl acetate (aq.) followed by lead citrate and observed with a Hitachi 7600 TEM at 80 kV. Images were captured with an AMT CCD XR80 (8-megapixel camera—side mount AMT XR80—high-resolution high-speed camera).

### Fluorescence labeling

Fluorescence labeling of cells was performed by seeding 5.0 × 10^4^ 4T1 cells in a 35 mm dish on a collagen-coated No. 1.5 coverslip with a 14 mm glass diameter (MatTek, Ashland, MA). After overnight incubation, the cells were washed once with serum-free media and replaced with fresh serum-free media. For cell cytosol labeling, Calcein-AM (Thermo Fisher Scientific, C1430) was added at a final concentration of 5 µg/mL and incubated for 10 min. For mitochondrial membrane labeling, JC-1 (Thermo Fisher Scientific, T3168) was added at a final concentration of 10 µg/mL and incubated for 10 min. To monitor the activity of caspases, CellEvent Caspase-3/7 Red detection reagents (Thermo Fisher Scientific, C10423) were used following the manufacturer’s instructions. For nuclear staining, Hoechst 33342 (Cell Signaling Technology, #4082) was added at a final concentration of 5 µg/mL and incubated for 10 min.

### Fluorescence microscopy

Cells were imaged on either a Zeiss LSM 700 or a 3i spinning disk confocal microscope. Samples were imaged with optimized microscope settings. Z-stack, time-lapse, or multi-channel acquisition settings were adjusted based on the experimental requirements. Images were then acquired with the appropriate excitation and emission wavelengths for each fluorophore.

### Image processing/analysis

Image processing was performed using Fiji software. For each image/video, the brightness and the contrast were adjusted. The time stamp and the scale bar were added. For quantification, CellProfiler was used. After cell segmentation based on the nucleus, the intensity of the signal of interest was quantified for each cell. Based on the intensity, each cells were labeled as positive or negative and the fraction of positive cells was calculated.

### Caspase-3 activity assay

Ten thousand 4T1 cells were seeded in 96-well plates and incubated overnight. After CKS1 treatment, the cells were lysed using a lysis buffer (Cell Signaling Technology, #7018). The activity of caspase-3 in the cell lysate was quantified using the caspase-3 activity assay kit (Cell Signaling Technology, #5723) following the manufacturer’s instructions. Briefly, the cell lysate was incubated with a substrate of caspase-3 which was designed to emit fluorescence when cleaved. After incubation, the fluorescence was measured using a plate reader. The protein concentration of the cell lysate was quantified using the DC protein assay kit (Bio-Rad). The caspase-3 activity was divided by the protein concentration to obtain the caspase-3 activity per µg/mL protein in the cell lysate.

### Quantification of ATP release

Two-lakh cells of 4T1 cells or CT26 cells in growth media were seeded in wells of a 96-well plate. After 12 h, the media was replaced with serum-free media, and the cells were treated with the corresponding concentration of CKS1. The cells were treated with CKS1 for 6 h and the supernatant was collected from each well. After filtering the supernatant with a 0.2 µm pore filter (Corning, 431229), the amount of ATP in the supernatant was quantified using the ENLITEN ATP assay system (Promega Corporation, Madison, WI, FF2000). We followed the instructions from the manufacturer when using the ENLITEN ATP assay system.

### Quantification of HMGB-1 release

Fifteen thousand cells of 4T1 cells or CT26 cells in growth media were seeded in wells of a 96-well plate. After 12 h, the media was replaced with serum-free media, and the cells were treated with the corresponding concentration of CKS1. The cells were treated with CKS1 for 6 h and the supernatant was collected from each well. After filtering the supernatant with a 0.2 µm pore filter (Corning, 431229), the amount of HMGB-1 in the supernatant was quantified using the Mouse/Rat HMGB1 ELISA kit (arigo biolaboratories, ARG81310).

### Immunogenicity experiments using DC2.4 cells

Two-lakh cells of 4T1 cells were seeded in wells of a 6-well plate. After overnight incubation, the media was replaced with 500 µL of fresh media, and 4T1 cells were treated with either 100 µM CKS1 or water for 2 h. In a separate well, 500 µL of fresh media was added and 100 µM CKS1 was incubated for 2 h. After the 2 h of incubation, all the supernatant was collected, filtered with 0.22 µm syringe filter, and was stored at −80 °C. Five lakh cells of DC2.4 cells were seeded in wells of a 24-well plate. After overnight incubation, the media was replaced with fresh media, supernatant from 4T1 cells treated with water, supernatant from 4T1 cells treated with 100 µM CKS1, or media containing 100 µM CKS1. DC2.4 cells were incubated for 2 h before RNA extraction.

### RNA extraction/reverse transcription

RNA extraction was conducted using QIAwave RNA Mini Kit (Qiagen, Germany, 74535) following the manufacturer’s instructions. Reverse transcription was conducted using a high-capacity cDNA reverse transcription kit (Thermo Fisher Scientific, 4368814) following the manufacturer’s instructions.

### Real-time quantitative PCR (qPCR)

Real-time qPCR was conducted using PowerUp SYBR Green Master Mix for qPCR (Thermo Fisher Scientific, A25741) following the manufacturer’s instructions. The following oligomers were produced by Genscript and were used as primers. GAPDH (GGACTTACAGAGGTCCGCTT and CTATAGGGCCTGGGTCAGTG), TNF (CTCATGCACCACCATCAAGG and ACCTGACCACTCTCCCTTTG), IL-6 (CTCTGGCGGAGCTATTGAGA and AAGTCTCCTGCGTGGAGAAA), IL-12b (GCACCAGCTTCTTCATCAGG and GGCAGACATCGTCTTTGCTT). QuantStudio 12 K Flex Real-Time PCR System (Thermo Fisher Scientific) was used to conduct real-time qPCR. The relative quantity of TNF, IL-6, and IL-12b was normalized to the relative quantity of GAPDH. Finally, the expression level of the cytokines was normalized to the control sample and the fold change was calculated.

### H&E staining

Tumors were fixed by immersion in 10% neutral buffered formalin (Millipore Sigma) for 48 h and subsequently dehydrated by immersion in 70%, 90%, and 100% ethanol followed by xylene for two changes of 30 min each. Tissues were embedded in paraffin blocks at 58 °C and sectioned at 4 μm using a microtome. Sections were floated in a water bath at 56 °C, embedded onto glass slides, and dried overnight. Next, sections were rehydrated by immersion in xylene, followed by 100%, 95%, 70% ethanol, and finally water for two changes of 5 min each. For staining, slides were immersed in Gill’s Hematoxylin #2 (Millipore Sigma) for 1 min, immediately rinsed in tap water to prevent overstaining, and immersed in Eosin Y (Millipore Sigma) for 1 min and rinsed. Finally, slides were again dehydrated, mounted with Citramount, coverslipped, and scanned at 40× with a NanoZoomer slide scanner.

### Statistics

All statistics analyses were performed using R. Differences between the two groups were determined using unpaired two-tailed Welch’s *t*-test and were considered statistically significant when *p* < 0.05. To determine the difference between the control group and the treatment groups, we used Dunnett’s test. We used two-way repeated-measures ANOVA to determine the significance of the treatment in the in vivo experiment monitoring tumor growth. All data were replicated in at least three independent experiments and the results are expressed as mean ± standard error from the mean (SEM). For experiments with small sample sizes, the results of each experiment were plotted. For experiments with significant variability between experiments, the representative data was shown with technical replicates plotted if applicable.

### Supplementary information

Supplemental video 1: CKS1 accumulates on the surface of cancer cells and enters the cells.

Supplemental video 2: CKS1 accumulates on the surface of cancer cells and enters the cells.

Supplemental video 3: CKS1 accumulates on the surface of cancer cells and enters the cells.

Supplemental video 4: CKS1 induces an influx of surrounding media, cell swelling, and a burst of cell membrane.

Supplemental video 5: CKS1 induces an influx of surrounding media, cell swelling, and a burst of cell membrane.

Supplemental video 6: CKS1 rapidly diminishes the mitochondrial membrane potential.

Supplemental video 7: CKS1 rapidly diminishes the mitochondrial membrane potential.

Supplemental data legends

Fig. S1: CKS1 induces rapid cell death in multiple cancer cell lines but not in non-cancerous cell lines.

Fig. S2: CKS1 induces rapid cell death in multiple cancer cell lines but not in non-cancerous cell lines.

Fig. S3: CKS1 diminishes mitochondrial membrane potential and activates the apoptotic pathway.

Fig. S4: Schematic image of how CKS1 induces cancer cell death.

## Data Availability

The data supporting the findings of this study are available from the corresponding author upon reasonable request.
