# Supplementary material for: Chemokine-derived oncolytic peptide induces immunogenic cancer cell death and significantly suppresses tumor growth
Source: Cell Death Discov. 2024 Apr 2;10:161. doi: 10.1038/s41420-024-01932-5 (PMC10987543; doi:10.1038/s41420-024-01932-5)
Supplement: Supplementary file 8 — Supplemental data legends [file 41420_2024_1932_MOESM8_ESM.docx]

**Supplemental data legends**

**Fig. S1: CKS1 induces rapid cell death in multiple cancer cell lines but not in non-cancerous cell lines.** (**A, B**) U2OS cells and MCA205 cells were treated with the indicated concentration of CKS1 for 6 hours. The release of LDH in the supernatant was measured. (**C**) HUVECs were treated with CKS1 and the incorporation of BrdU was measured. Means ± SEM, N = 3, Dunnett’s test (***p < 0.005).

**Fig. S2: CKS1 induces rapid cell death in multiple cancer cell lines but not in non-cancerous cell lines.** (**A**) 4T1 cells were pre-treated with 50 µM pan-caspase inhibitor Z-VAD for 30 minutes or 50 µM RIPK1 inhibitor Nec-1s for 30 minutes. After the pre-treatment, 4T1 cells were treated with 100 µM CKS1 for 6 hours. The release of LDH in the supernatant was measured by the CyQUANT LDH cytotoxicity assay and was normalized to the amount of LDH released from cells treated with lysis buffer. Means ± SEM, N = 3, Dunnett’s test (***p < 0.005). (**B**) 4T1 cells were pre-treated with 1 µM Fer-1 for 30 minutes and treated with 100 µM CKS1 for 6 hours. The release of LDH in the supernatant was measured and normalized. Means ± SEM, N = 3, Dunnett’s test (***p < 0.005). (**C**) 4T1 cells stained with calcein-AM were pre-treated with 50 µM Z-VAD for 30 minutes. The cells were treated with 100 µM CKS1 and were observed by live cell imaging. The ratio of cells maintaining calcein in the cytosol at each time point was calculated based on the live cell imaging. Representative of N = 3.

**Fig. S3: CKS1 diminishes mitochondrial membrane potential and activates the apoptotic pathway.** (**A**) 4T1 cells were treated with 100 µM CKS1 for the indicated time. The activity of caspase-3 was quantified by caspase-3 assay. The measured fluorescence was divided by the protein concentration in the cell lysate. Means ± SEM, N = 3, Dunnett’s test (*p < 0.05). (**B**) Representative images of 4T1 cells stained with CellEvent Caspase-3/7 Red detection reagents and treated with 100 µM CKS1.

**Fig. S4: Schematic image of how CKS1 induces cancer cell death.** First, the peptides accumulate on the cell surface of cancer cells. Next, the peptides form pores on the cell surface and enter the cell. If the cell membrane damage is severe, the cells will be lysed. If the cells survive after the initial damage to the cell membrane, the apoptotic pathway will be activated.

**Supplemental video 1: CKS1 accumulates on the surface of cancer cells and enters the cells.** 4T1 cells were treated with 100 µM of FITC-tagged CKS1 and were observed by a confocal microscope. The video shows one frame taken every 10 seconds. The number on the left-top corner indicates the time after FITC-tagged CKS1 was treated.

**Supplemental video 2: CKS1 accumulates on the surface of cancer cells and enters the cells.** The zoomed-out version of supplemental video 1.

**Supplemental video 3: CKS1 accumulates on the surface of cancer cells and enters the cells.** CT26 cells were treated with 100 µM of FITC-tagged CKS1 and were observed by a confocal microscope. The video shows one frame taken every 30 seconds. The number on the left-top corner indicates the time after FITC-tagged CKS1 was treated.

**Supplemental video 4: CKS1 induces an influx of surrounding media, cell swelling, and a burst of cell membrane.** 4T1 cells were stained with calcein-AM and treated with 100 µM CKS1. The morphology of the cells was observed by a confocal microscope. The video shows one frame taken every 10 seconds. The number on the left-top corner indicates the time after CKS1 was treated.

**Supplemental video 5: CKS1 induces an influx of surrounding media, cell swelling, and a burst of cell membrane.** 4T1 cells were stained with calcein-AM and Hoechst 33342. The morphology of the cells was observed by an epifluorescent microscope. The video shows one frame taken every 10 seconds. The number on the left-top corner indicates the time after CKS1 was treated.

**Supplemental video 6: CKS1 rapidly diminishes the mitochondrial membrane potential.** 4T1 cells were stained with JC-1 and treated with 100 µM CKS1. The red signal indicates the aggregate form of JC-1, which appears when the mitochondrial membrane potential is high. The green signal indicates the monomeric form of JC-1, which appears when the mitochondrial membrane potential is low. The video shows one frame taken every minute. The number on the left-top corner indicates the time after CKS1 was treated. The negative numbers indicate pre-treatment, and CKS1 was treated at time 0.

**Supplemental video 7: CKS1 rapidly diminishes the mitochondrial membrane potential.** The zoomed-out version of supplemental video 6.
