# Supplementary figures and images for: Chemokine-derived oncolytic peptide induces immunogenic cancer cell death and significantly suppresses tumor growth
Source: Cell Death Discov. 2024 Apr 2;10:161. doi: 10.1038/s41420-024-01932-5 (PMC10987543; doi:10.1038/s41420-024-01932-5)

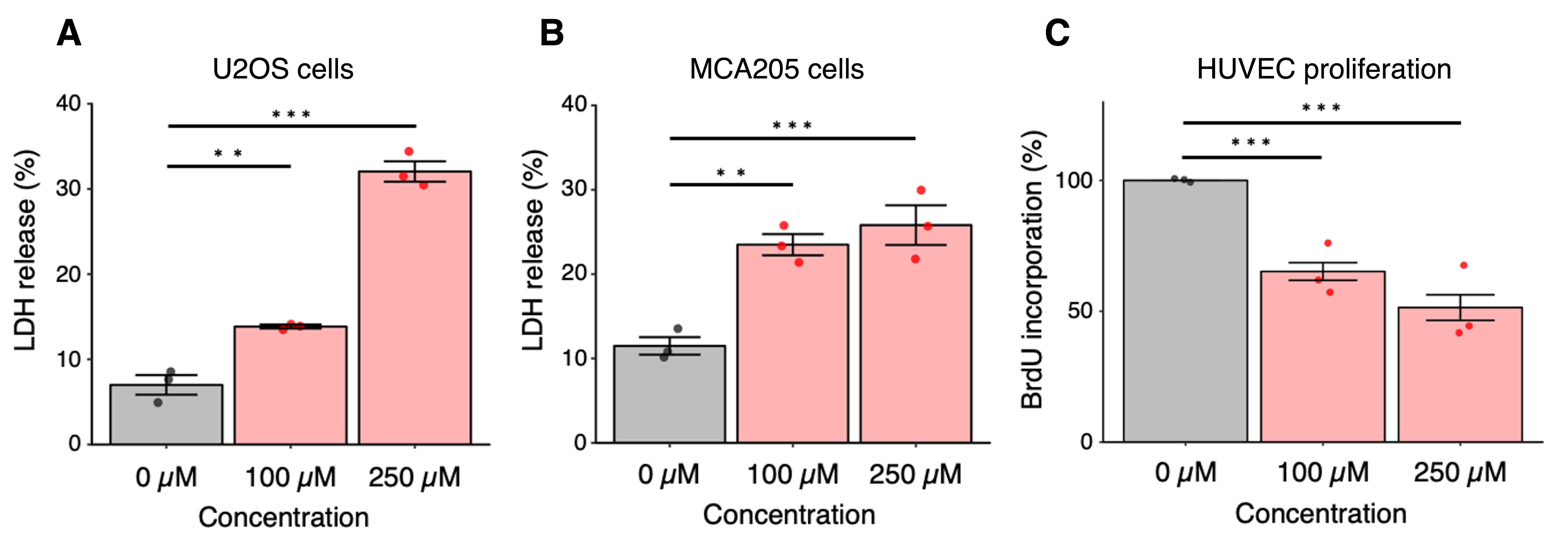

Supplement: Supplementary file 9 — Fig. S1: CKS1 induces rapid cell death in multiple cancer cell lines but not in non-cancerous cell lines. [file 41420_2024_1932_MOESM9_ESM.png]

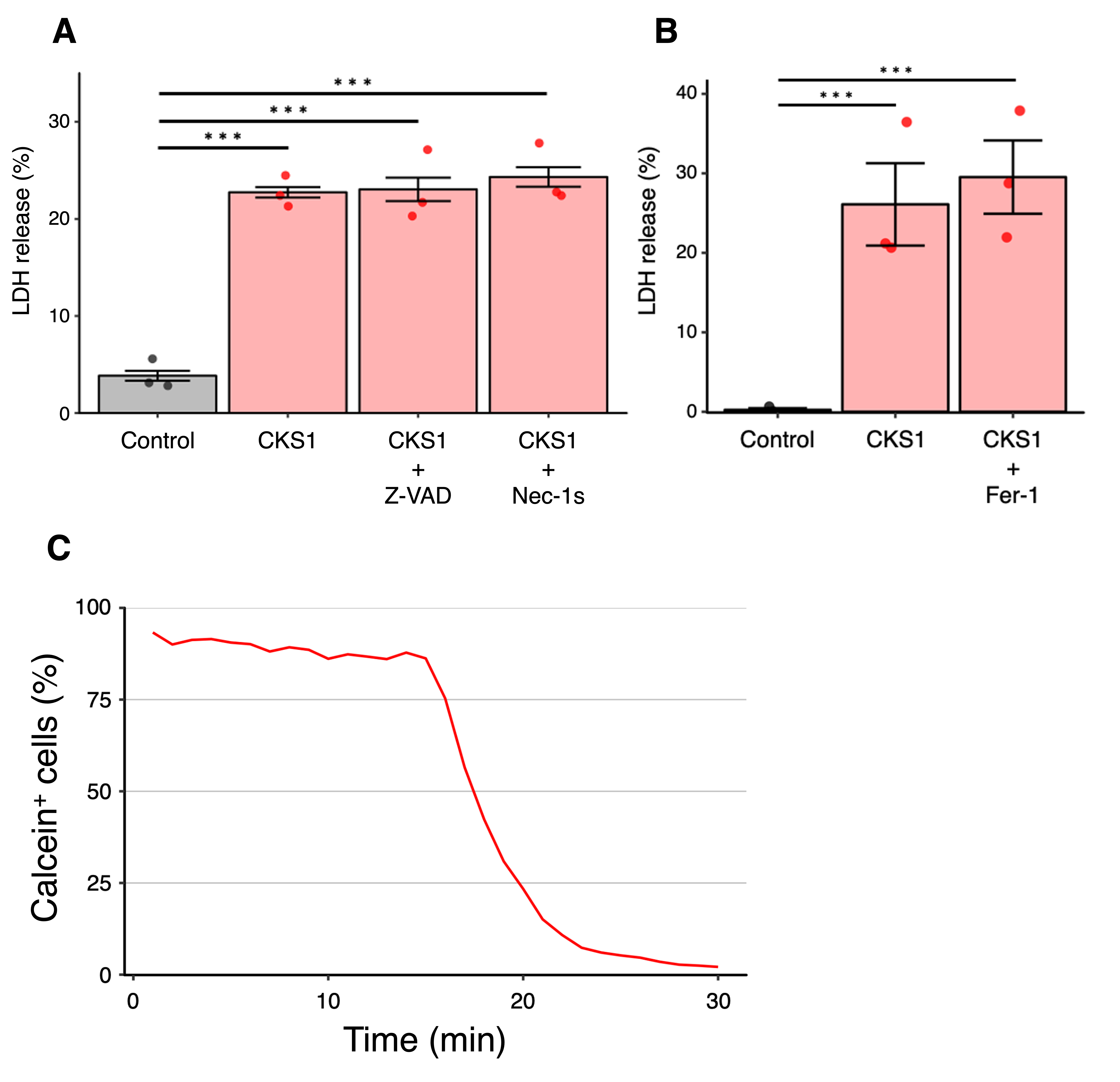

Supplement: Supplementary file 10 — Fig. S2: CKS1 induces rapid cell death in multiple cancer cell lines but not in non-cancerous cell lines. [file 41420_2024_1932_MOESM10_ESM.png]

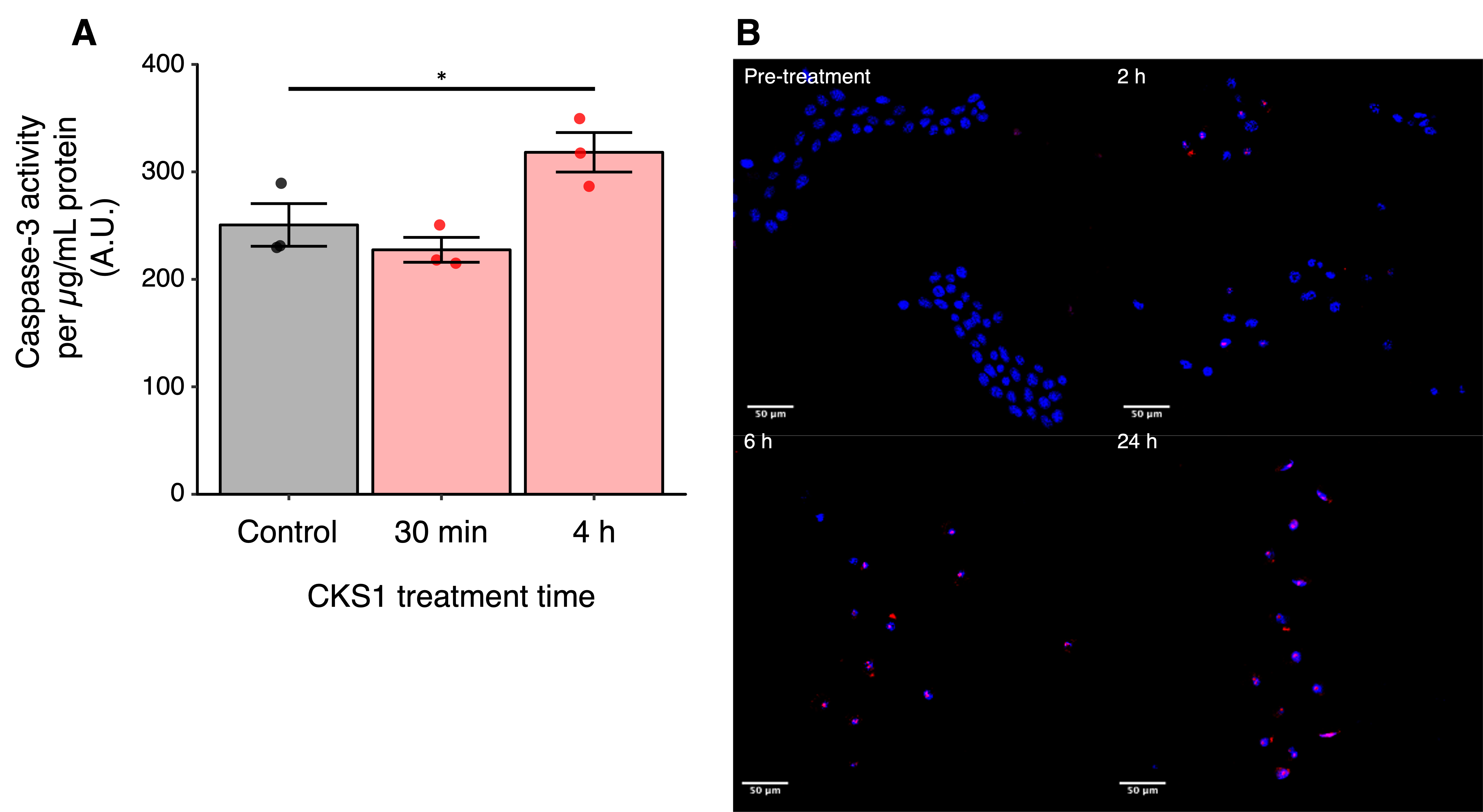

Supplement: Supplementary file 11 — Fig. S3: CKS1 diminishes mitochondrial membrane potential and activates the apoptotic pathway. [file 41420_2024_1932_MOESM11_ESM.png]

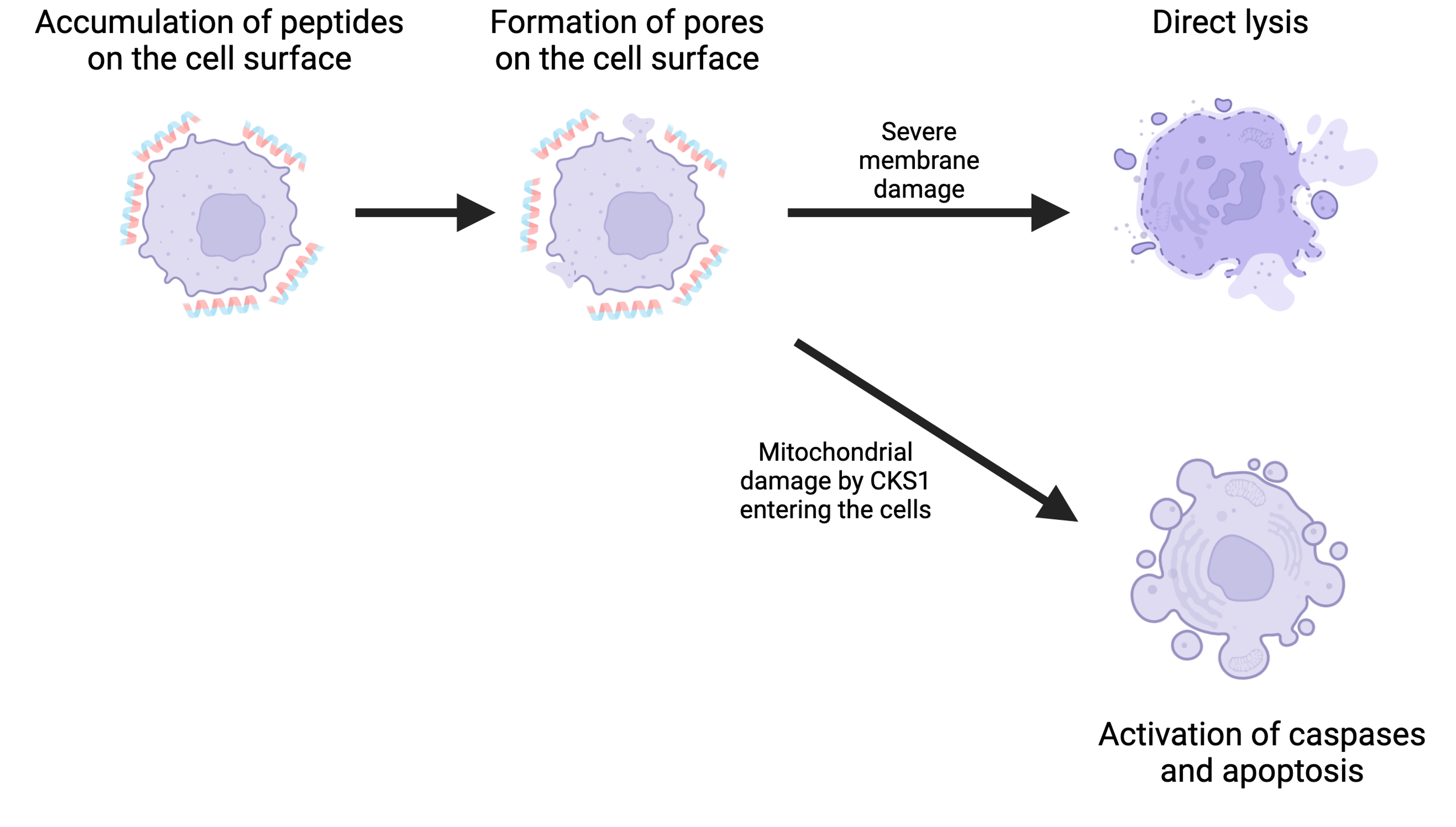

Supplement: Supplementary file 12 — Fig. S4: Schematic image of how CKS1 induces cancer cell death. [file 41420_2024_1932_MOESM12_ESM.png]
